# Annotations

**Published:** 1888-12-29

**Authors:** 


### ANNOTATIONS.

"Let him die."

"THE old year is dying: let him die," wrote Tennyson a generation ago. The mere words sound harsh, as if the poet had held a grudge in his heart against the old year. Poets, like other persons, or perhaps even more than other persons, do cherish grudges, and not seldom bring them out to public view at unfortunate times. Perhaps, however, *De mortuis* does not apply to old years. Anybody may say what he pleases of them, be it good, bad, or indifferent, without offence. Nevertheless, human nature sometimes indulges in mere sentiment—sentiment that has no particular connexion with any palpable fact or circumstance, and human nature is none the worse for a little of such vague and gentle nursing. The old year is very nearly dead as we write. How many men and women, hopes and ambitions, fears and anxieties have died with him! To some he has been worse than he promised at the beginning; to others better. The burdens of some have been piled up month after month until it seemed as if the load must crush them beyond hope of recovery. From the shoulders of others weight after weight and care after care have been removed. Old cases of long standing, new ones of yesterday, both have alike been diminished or taken away, and the cold days of December and January are as the spring-tide and the summer. There is only one way of satisfactorily explaining either an increase or a diminution of care. Let it be seen that each is disciplinary, that each may be made to serve the high purpose of training and mental and moral development, then the mind can be satisfied, and hope can live. But if all the events of life, great and small, are alike attributed to the blindest of chances, the most fortuitous of accidents, then the piling-up of misfortunes becomes the death and extinction of hope, of courage, and of character alike. "The old year is dying: Let him die," and let there pass out with him into the eternal shades everything that makes man or woman less than noble, less than human. Let feebleness of purpose disappear; let doublemindedness vanish; let selfishness, the worst bane of manhood and womanhood, be ruthlessly driven after them. Let there come in with the new year that singleness of eye, that simplicity of mind which religion and true science alike love. Every man and woman who has reached the age of forty must have discovered that feeble purposes and inconsequent living lead to dissatisfaction and self-contempt. With the new year new purposes should be made and life should be resolutely lived on the only level which is worthy of the intellect and the soul of man. "Ring out the old; ring in the new; ring out the false; ring in the true......Ring in the good that is to be."

Hospitals at Home.

THE South London District Nursing Association is applying to the Hospital Sunday Fund for participation in next year's collection. In the petition, which is addressed to the Lord Mayor, as Chairman, the reasons are set forth at length by which the Association justifies its claim. Those who know the institution and its work will probably think that there is no need for a lengthened and elaborate plea; but they must remember that it is a new departure which is proposed to be taken, and new departures which involve the expenditure of public money, or the interference, in however small a degree, with vested interests, must always be asked to show cause. For our part, we have not the slightest hesitation in fully and freely endorsing the prayer of the Association. Almost every plea that can be put forth by the promoters of hospitals on behalf of the support of their institutions will hold good for nursing associations such as this; and some pleas may be offered which are even stronger than most of those urged by hospitals. The District Nursing Association labours among the sick poor; its services are gratuitous; the nurses are all trained ladies; they only work under the instructions of properly qualified medical men; their aid enables cases to be treated at home, and so saves the funds of the hospital. They attend cases which cannot be taken to hospitals, such as puerperal fever; their influence tends to spread the knowledge and the practical observance of sanitary laws. It is also pointed out that the fund is no longer limited to hospitals, but is given to dispensaries and to convalescent homes therefore, say the South London Association "why not to us?" Why not, indeed? There are nine solid and substantial reasons in favour of the proposal. We cannot imagine one opposing argument which can weigh down all these, or even a small portion of them. The reasons in favour are absolutely convincing. So far as we are concerned, we should not have thought it necessary to state them. The work of the Association is entirely similar to that of hospitals, and in many respects identical. The provinces are not seldom ahead of London in new departures of this kind. The Manchester Hospital Sunday Fund has already considered the subject, and has decided to vote a first grant of £250 to the District Nursing Association. It need not be anticipated that the managers of the London Fund will raise any serious obstacles. They are men of wide sympathies and of a sound knowledge of general business principles. The South London Association, it may be hoped, will steadily and wisely prosecute their petition, and be rewarded with the success which their good work so well deserves.

A Painful Warning.

MANY persons have the habit of popping things into their mouths, for want of any receptacle more immediately convenient; and many others, who are too careless to keep a small pair of scissors at hand, use their teeth for biting purposes, especially when threading needles or sewing. These practices are always dangerous, and every now and again some catastrophe occurs which shows in a most painful way how easy it is for death to result from simple carelessness and want of thought. Mr. Hicks, the coroner, held an inquiry a few days ago at St. Bartholomew's Hospital on the dead body of Alfred Thomas Smith, a schoolboy, twelve years old. On November 22nd, Smith, when at school, bit the point off a nib he had in his hand, and the remainder slipped down his throat. He consulted a medical man, and also went to a hospital; but nothing being discovered, he was advised to eat plenty of stale bread—for what purpose is not very evident. Two days after he had severe pains in the neck, and was decidedly ill. Thereupon he was removed to St. Bartholomew's Hospital, where he remained until he died. The points to be borne in mind here are that this boy had the very best skill and care which the medical science of the time affords, and yet that he died from merely swallowing a portion of a pen. It is not for a moment to be supposed that there was any want of competency on the part of the physicians in charge. On the contrary, the unusual and interesting nature of the case would prompt them to try any imaginable means of saving the boy's life. Eight days after the accident he vomited the pen; but the mischief had then been done. The pen had got fixed in the larynx at the outset, and no efforts had been successful in dislodging it. There it had set up ulceration and suppuration. Bleeding had followed later; and the exhaustion resulting from the ulcerative process, together with the bleeding, caused death in three or four weeks. It is the custom of railway companies to post up at stations little notices proclaiming that on such a date, John Blank had done certain injuries to the company, and had been fined or sent to prison. These notices are put up to warn all other passengers against like misdeeds. It would be well if every school where boys bite pens and other things and every work-room where women dine off thread-ends with infinite mischief to digestion and danger to life, could have this and similar cases placarded for general warning. This particular instance is recorded in the public prints; but many almost as serious are happening constantly, though they are never heard of. Want of care often does more harm than want of knowledge.

